# Pannexin 3 ER Ca^2+^ channel gating is regulated by phosphorylation at the Serine 68 residue in osteoblast differentiation

**DOI:** 10.1038/s41598-019-55371-9

**Published:** 2019-12-10

**Authors:** Masaki Ishikawa, Geneva Williams, Patricia Forcinito, Momoko Ishikawa, Ryan J. Petrie, Kan Saito, Satoshi Fukumoto, Yoshihiko Yamada

**Affiliations:** 1 Operative Dentistry, Tohoku University Graduate School of Dentistry, Sendai, 980-8575 Japan; 2 Molecular Biology Section, National Institute of Dental and Craniofacial Research, National Institutes of Health, Philadelphia, USA; 3 Department of Pediatric Dentistry, Tohoku University Graduate School of Dentistry, Sendai, 980-8576 Japan; 4 Department of Biology, Drexel University, Philadelphia, PA 19104 USA

**Keywords:** Differentiation, Ion channel signalling, Bone development, Calcium signalling

## Abstract

Pannexin 3 (Panx3) is a regulator of bone formation. Panx3 forms three distinct functional channels: hemichannels, gap junctions, and endoplasmic reticulum (ER) Ca^2+^ channels. However, the gating mechanisms of the Panx3 channels remain unclear. Here, we show that the Panx3 ER Ca^2+^ channel is modulated by phosphorylation of the serine 68 residue (Ser68) to promote osteoblast differentiation. Among the 17 candidate phosphorylation sites identified, the mutation of Ser68 to Ala (Ser68Ala) was sufficient to inhibit Panx3-mediated osteoblast differentiation via reduction of Osterix and ALP expression. Using a Ser68 phospho-specific antibody (P-Panx3) revealed Panx3 was phosphorylated in prehypertrophic, hypertrophic chondrocytes, and bone areas of the newborn growth plate. In osteogenic C2C12 cells, P-Panx3 was located on the ER membranes. Importantly, the Ser68Ala mutation only affected Panx3 ER Ca^2+^ channel function. Ser68 on Panx3 was phosphorylated by ATP stimulation and PI3K/Akt signaling. Finally, real-time FRET imaging and ratio analysis revealed that the Panx3 channel conformation was sensitive to ATP. Together, the phosphorylation of Panx3 at Ser68 is an essential step controlling the gating of the Panx3 ER Ca^2+^ channel to promote osteogenesis.

## Introduction

Intracellular Ca^2+^ signaling regulates cell proliferation, differentiation, morphology, and functions^1^. Intracellular Ca^2+^ levels ([Ca^2+^]_i_) is controlled by the extracellular space and the ER, a Ca^2+^ storage organelle. Ca^2+^ is released from the ER through ER Ca^2+^ channels located on the ER membrane. These receptors include the inositol-3-trisphosphate (IP3) receptors (IP3Rs) that are ubiquitously expressed^2^ and the ryanodine receptors (RyRs) expressed in some tissues^3^. Additionally, pannexins (Panxs), which are members of the gap junction protein family, were recently reported to act as ER Ca^2+^ channels. The Panx1 ER Ca^2+^ channel functions in prostate cancer cells^4^, and Panx3 acts as an ER Ca^2+^ channel in osteoblasts^5^.

Gap junctions regulate various intracellular and physiological functions via the exchange of small molecules, such as ATP and ions, from the cell to the extracellular space via hemichannels and from cell to cell via gap junctions^6–9^. Vertebrates have two gap junction protein families: the connexins (Cxs) and the pannexins (Panxs). The Cxs have more than 20 members and their functions have been reported^10^. Dysregulations and mutations of Cxs are involved in many human diseases, including cancer, hypertension, atherosclerosis, and developmental abnormalities^11^. By contrast, Panxs are less well characterized and they consist of only three known members (Panx1–3)^12–14^. Panx1 is widely expressed, especially in the central nervous system, Panx2 is frequently expressed in the central nervous system^15^, and Panx3 expression has been reported in the skin, cochlea, and developing hard tissues, including cartilage, bones, and teeth^5,16–20^. The human disorders linked to the Panxs, and especially Panx1, are associated with ischemia, stroke, overactive bladder, HIV infections, Crohn’s disease, and platelet aggregation^21^. Further, a germline variant in *PANX1* has been associated with dysfunctions that included intellectual disabilities, hearing loss, and other multisystem failures^22^. Panx3 has been linked to osteoarthritis (OA), a disabling degenerative joint disorder with cartilage destruction, subchondral bone remodeling, and inflammation of the synovial membrane^23^.

Panx3 is now recognized as a new regulator of bone growth^24^. Previously, we have identified that Panx3 promotes chondrocyte differentiation by the ATP released via the Panx3 hemichannel, which counteracts the parathyroid hormone (PTH)–related protein (PTHrP) signaling pathway^16^. We also reported that Panx3 promotes osteoblast differentiation via its functions as a hemichannel, an ER Ca^2+^ channel, and a gap junction^5^. In addition, Panx3 regulates the osteoprogenitor cell cycle exit by inhibiting Wnt/β-catenin signaling through its hemichannel^25^. *Panx3*-null (*Panx3*^*−/−*^) mice show low bone density and marked dwarfism caused by the failure in both endochondral and intramembranous ossification. An *in vivo* study showed that Panx3 regulates mature hypertrophic chondrocyte differentiation and is requred in osteogenesis from the early stage, whereas Cx43 plays a role in the maturation stage. We also demonstrated that Panx3 and Cx43 play distinct roles in bone formation^26^.

Both Cxs and Panxs have common protein structures, including four transmembrane domains, two extracellular loops, one intracellular loop, and N- and C-terminal segments^10,27^. The tetramer of the subunit forms a channel structure that functions as a hemichannel, gap junction, and ER Ca^2+^ channel, and the ER Ca^2+^ channel is Panxs specific. Recently, Panx1 and Panx3 were recognized as N-linked glycosylate proteins. Panx1 has the glycosylation site at asparagine 254 in the second extracellular loop, on the other hand, Panx3 at asparagine 71 in the first extracellular loop^28^. Panx2 also contains a potential N-linked glycosylation consensus site at asparagine 86, although the glycosylation of this residue has not yet been confirmed. Glycosylation of Panxs plays a role in the appropriate trafficking of these Panxs to the cell surface^18,29^. However, the mechanisms controling the opening or closing of Panxs, and especially the Panx3 channel, are not yet understood.

In this study, we showed that the Panx3 ER Ca^2+^ channel is activated by phosphorylation at the Ser68 residue by ATP-mediated PI3K/Akt signaling to promote osteoblast differentiation. Phosphorylation of Panx3 at Ser68 increases intracellular Ca^2+^ levels through Panx3 ER Ca^2+^ channel gating, but not via its hemichannel or gap junction functions. Our results reveal that the Panx3 ER Ca^2+^ channel is regulated by a distinct gating mechanism that differs from the mechanism regulating the hemichannel and gap junction functions.

## Results

**Figure 1:**
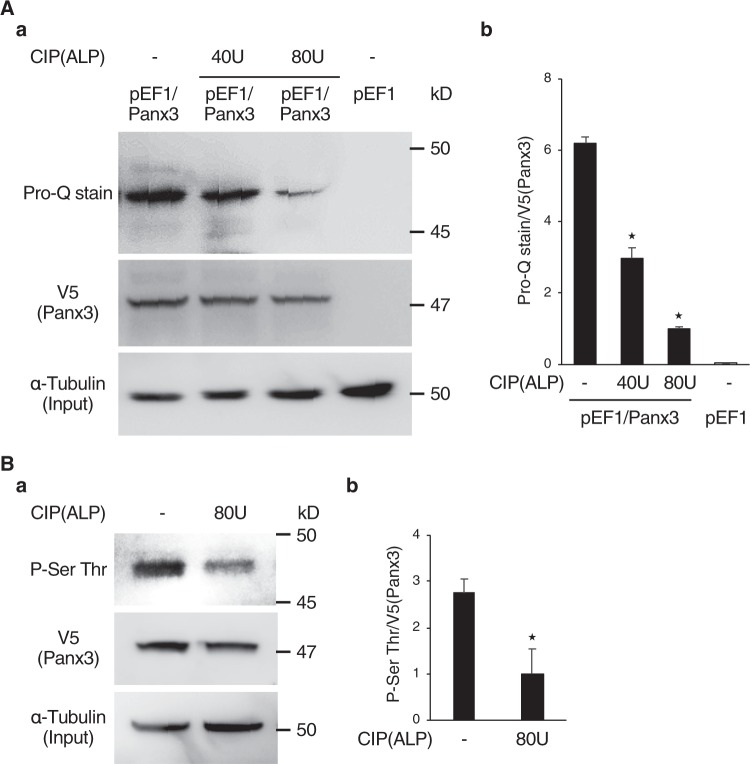
Panx3 is phosphorylated. (**A**,a) A phosphorylation band was revealed from pEF1/Panx3 cell lysates by Pro-Q Diamond phosphoprotein gel staining. Control (pEF1) cells and C2C12 cells transiently transfected with Panx3 (pEF1/Panx3) were cultured for 2 days in DMEM containing 10% FBS. Immunoprecipitated cell lysates were loaded onto NuPAGE Bis-Tris gels. After electrophoresis, the gels were fixed and stained with Pro-Q stain. Lysates were incubated with CIP (ALP:40 or 80 U) for 45 min at 30 °C before loading the gel. Lysates were analyzed by western blotting with a α-tubulin as a control, to confirm loading of the same amount of protein (bottom). (b) Quantification of the ratios of Pro-Q stained band/V5. (**B**,a) Immunoprecipitation assays with cell lysates from pEF1/Panx3 transiently transfected cells showed a phosphorylation band of serine and threonine which was reduced by CIP. The Panx3 protein overexpressed in C2C12 cells was precipitated with anti-V5 antibody and analyzed by immunoblotting with Phospho-Serine and Threonine (P-Ser Thr) antibody. Precipitated lysates were treated with CIP (80U) before loading. (b) Quantification of the ratios of P-Ser Thr/V5. *P < 0.01. Error bars represent the mean ± SD; *n* = 3. Pro-Q stain and Western blot were perfomed by at least three independent experiments for each experimental group. Full blot is shown in the Supplemental Information (Full Original Blots-I).

We analyzed the mechanisms of Panx3 channel gating by first testing whether Panx3 is phosphorylated using Panx3 overexpressing C2C12 cells cultured in osteogenic media by Pro-Q diamond phosphoprotein gel staining^30,31^, and immunoprecipitation assays (IP). Pro-Q diamond phosphoprotein gel-staining methods were used: total Panx3 protein was immunoprecipitated with V5 antibody, followed by detection of phosphorylation with the Pro-Q gel-staining method. In both Pro-Q staining and IP, total Panx3 protein in immunoprecipitated cell lysate was detected by Western blot using V5 antibody. The amount of total extracted protein (Input) was confirmed with α-tubulin antibody. Cell lysates from the Panx3 overexpressing cells showed a phosphorylated band similar in size to the Panx3 molecular weight, 47 kD, after Pro-Q staining (Fig. 1A,a). The size of the phosphorylated band was dose-dependently decreased by treatment with CIP (ALP) phosphatase (Fig. 1A,a,b). Further, IP with the Panx3 protein showed that the phosphorylated band detected between 45 and 50 kD was recognized by an antibody for serine and threonine phosphorylation. The size of that phosphorylated band was also decreased after CIP treatment (Fig. 1B,a,b). These results suggested that Panx3 is phosphorylated in cultured cells.

**Figure 2:**
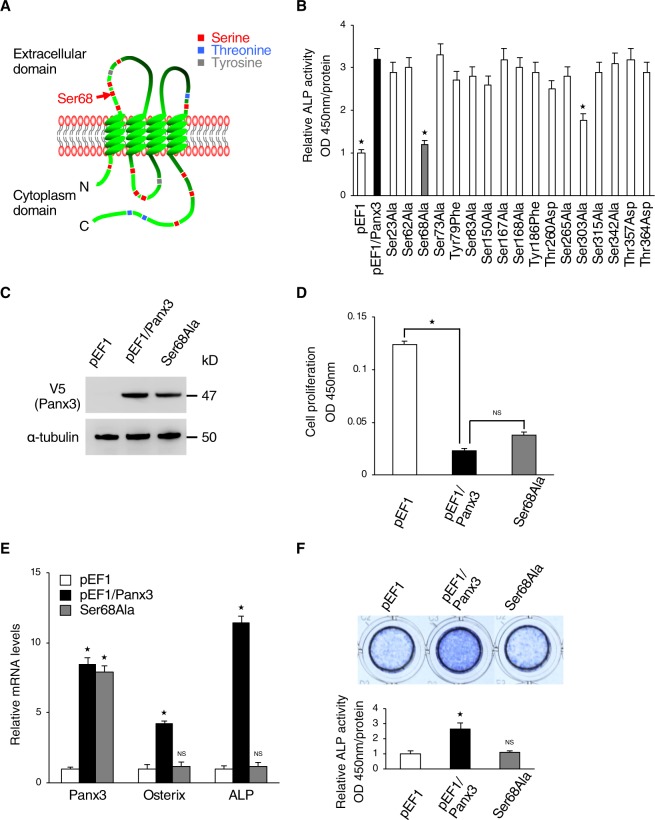
Ser68 is candidate phosphorylation residue for regulation of Panx3 function in osteoblast differentiation. (**A**) The locations of 17 candidate phosphorylation residues in the Panx3 protein. Ser68 (red arrow) is located in the first extracellular loop. (**B**) Screening of candidate sites of interest by ALP activity assays with mutation constructs in each of the 17 candidate sites. C2C12 cells transiently transfected with each mutation construct were cultured with DMEM + BMP2 (300 ng/ml) for 2 days. Normalized by each Panx3 protein expression detected by western blotting. (**C**) Establishment of Ser68Ala stably transfected C2C12 cells. Western blot of Panx3 expression in Ser68Ala cells compared with normal Panx3 stably transfected C2C12 cells (pEF1/Panx3). Western blot was perfomed by at least three independent experiments. Full blot is shown in the Supplemental Information (Full Original Blots-II). (**D**) Proliferation of pEF1/Panx3 or Ser68Ala stably transfected cells. Cells were cultured in DMEM media for 2 days. (**E**) C2C12 cells stably transfected with a control pEF1 vector, pEF1/Panx3 or Ser68Ala were cultured with BMP2 (300 ng/ml) for 3 days. Total RNA then was extracted and mRNA levels of Panx3 and the osteoblast differentiation marker, Osterix, and ALP were analyzed by quantitative RT-PCR. (**F**) Ser68Ala inhibited Panx3-promoted ALP activity. Representative ALP staining (top) and its quantitative data (bottom). Cells stably transfected with pEF1/Panx3 and Ser68 were cultured with BMP2 (300 ng/ml) for 3 days. *P < 0.01. NS, nonsignificant. Error bars represent the mean ± SD; *n* = 3.

We identified specific phosphorylation sites in Panx3 protein by first using a database (NetPhos 2.0 Server) to analyze the candidate sites. We found 17 phosphorylation candidates and made point mutation constructs of each Panx3 phosphorylation candidate site (Fig. 2A). We then used the ALP activity assay as a screen to test which mutation site affected Panx3-mediated osteoblast differentiation. Among the 17 candidate sites, the mutation of the serine 68 (Ser68) and serine 303 (Ser303) residue inhibited the Panx3-mediated promotion of ALP activity (Fig. 2B). Since the mutation of Ser68 revealed the highest inhbition, we then generated a stable cell line of osteogenic C2C12 cells transfected with a phosphorylation of Ser68 mutation (Ser68Ala) vector (Fig. 2C). The cell proliferation rate of the stable Ser68Ala cells was not different from that of the normal Panx3 stably expressed cells (Fig. 2D), whereas the Ser68Ala cells clearly showed reduced Panx3-mediated osterix and ALP expression by qPCR analysis and ALP staining after culturing in osteoinduction medium (Fig. 2E,F). These results indicated that the Ser68 residue of Panx3 was an important site for osteoblast differentiation, but not proliferation.

**Figure 3:**
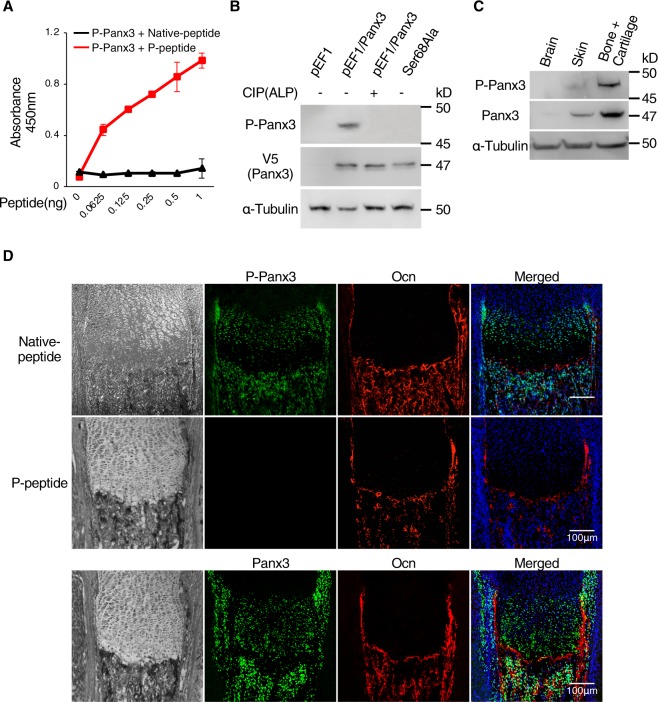
Generation of P-Panx3 specific antibody and its localization in the tibial growth plate of newborn mice. (**A**) Specificity evaluation of phosphorylated Panx3 at Ser68 antibody (P-Panx3) against antigen-peptide (P-peptide) or control (Native peptide) by ELISA. ELISA was performed in dishes coated with serial dilutions of P-Panx3 peptide. Error bars represent the mean ± SD; *n* = 6. (**B**) Western blots, using P-Panx3 antibody and lysates from C2C12 cells stably transfected with pEF1/Panx3 and Ser68Ala. Protein was isolated after 1 day of culture in DMEM. For dephosphorylation, protein from pEF1/Panx3 cells was treated CIP (80U) before loading. V5 antibody was used for detection of Panx3 expression. (**C**) Western blots using P-Panx3 and Panx3 antibodies and tissue lysates isolated from brain, skin and bone + cartilage of newborn mice. (**D**) Immunohistochemistry of newborn mouse growth plates. Image observed under light microscopy (black & white, left vertical lane), P-Panx3 (green, upper and middle lane), Panx3 (green, bottom lane), Ocn (red), Hoechst nuclear staining (blue) and merged image (right vertical lane) The middle lane is staining images of P-Panx3 in the presence of its antigen peptide (P-peptide). Western blot was perfomed by at least three independent experiments. Full blot is shown in the Supplemental Information (Full Original Blots-III).

We generated a phosphorylation-specific antibody to the Ser68 residue of Panx3 (P-Panx3) and tested its specificity with an ELISA assay. The P-Panx3 antibody specifically bound the antigen peptide (P-peptide) (Fig. 3A). We then used the P-Panx3 antibody and western blotting to determine whether Panx3 was phosphorylated *in vitro* at Ser68. The protein lysate from Panx3 expressing C2C12 cells showed the expected band of P-Panx3 between 45 and 50 kD molecular weight, and the size of that band was diminished by CIP treatment and in cell lysates from Ser68Ala transfected cells (Fig. 3B). These results indicated that the P-Panx3 antibody could detect Panx3 phosphorylation at Ser68. We also clearly detected P-Panx3 in skin, bone, and cartilage tissue from newborn mice by western blotting of molecular weights similar to those in cell culture, but its presence was unclear in brain (Fig. 3C). We immunostained the growth plates of newborn mice to elucidate the site where P-Panx3 was expressed. P-Panx3 was expressed in prehypertrophic and hypertrophic chondrocytes, the perichondrium/periosteum, and the bone area. Interestingly, the intensity of the positive P-Panx3 signal was higher in the prehypertrophic zone than in the proliferative and hypertrophic zones. The immunostaining signal of P-Panx3 disappeared following application of the P-Panx3 antibody mixed with P-peptide (Fig. 3D).

**Figure 4:**
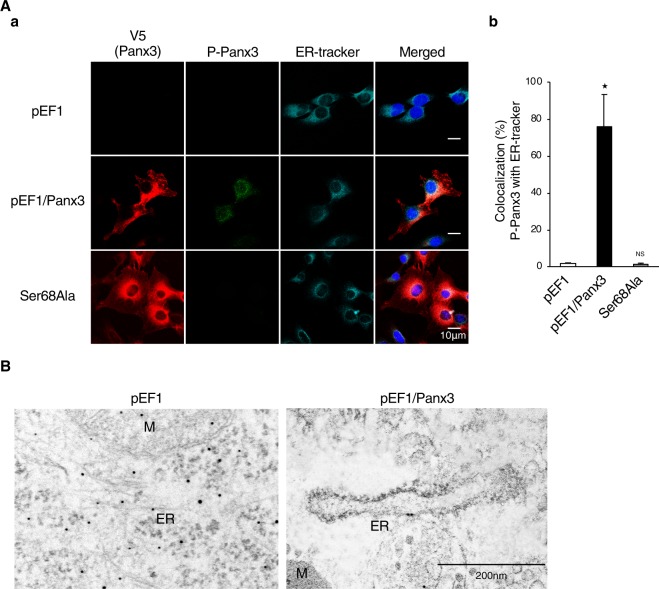
Phosphorylated Panx3 at Ser68 is localized on the ER membrane. (**A**,a) Cellular localization of P-Panx3 in cells stably transfected with pEF1, pEF1/Panx3, or Ser68Ala. Fluorescent confocal images showed Panx3 (red), P-Panx3 (green), ER-tracker (cyan), and Hoechst nuclear staining (blue). (b) Measurements show a percentage of colocalization between P-Panx3 with ER-tracker. (**B**) Immuno-TEM demonstrates P-Panx3 localization on the ER membrane in pEF1/Panx3 transfected cells (right panel). M: mitochondria, ER: endoplasmic reticulum. The micrographs shown are representative of 15–20 cells obtained from three independent experiments. *P < 0.01. NS, nonsignificant. Error bars represent the mean ± SD; *n* = 12.

The next question we addressed was how P-Panx3 regulates Panx3 channel functions. We first determined the cellular location of P-Panx3 expression by immunocytochemistry. The merged image obtained with P-Panx3 and ER-tracker or Panx3 revealed that P-Panx3 was expressed strongly on ER membranes, rather than on the plasma membrane or gap junction. Quantitative analysis revealed that 75% of the P-Panx3 protein was colocalized with ER tracker, while no P-Panx3 expression was observed in the Ser68Ala stably transfected C2C12 cells (Fig. 4A,a,b). Further, we also found that P-Panx3 was located on the ER membrane by transmission electron microscopy (TEM) after immunolabeling with P-Panx3 antibody (Fig. 4B). These results suggest that Panx3 is phosphorylated at the Ser68 residue on the ER membrane.

**Figure 5:**
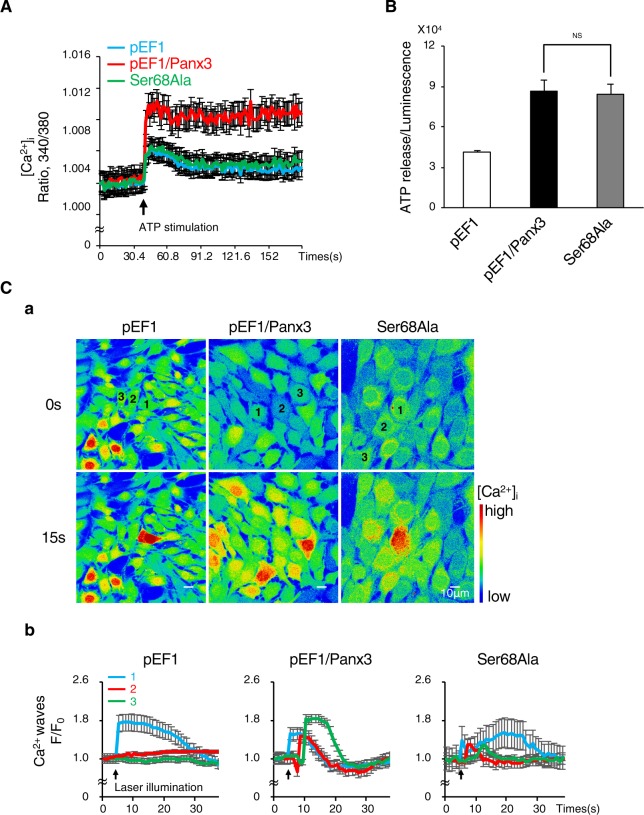
Phosphorylation at Ser68 regulates the functioning of the Panx3 ER Ca^2+^ channel, but not the hemichannel or gap junction. (**A**) ER Ca^2+^ channel activities in cells stably transfected with pEF1, pEF1/Panx3 or Ser68Ala. The increase in intracellular Ca^2+^ released from the ER by ATP stimulation (arrow) was measured. Average traces of at least four different experiments are shown with the solid lines. The arrow indicate the time of ATP stimulation. (**B**) Hemichannel activities in each stable cell line. ATP release into the extracellular space was measured for 2 min. NS, nonsignificant. (**C**) Gap junction activities in each stable cell line. (a) Representative images of the Fluo-4 fluorescence before stimulation (upper) and 15 s after the stimulation (bottom) and the fluorescence intensity traces of laser stimulated single cell (1), one (2) and two cell (3) distant to stimulated single cell (b). Average traces of at least three different experiments are shown with the solid lines. The arrow indicate the time of ATP stimulation. The Ca^2+^ wave was measured in cells loaded with Fluo-4 and NP-EGTA (caged Ca^2+^) by starting uncaging in a single cell using laser illumination. The Ca^2+^ wave propagation was measured at 30 s after the illumination. The images shown are representative of at least three different experiments.

The results of cellular localization of P-Panx3 predicted that Ser68 phosphorylation would play a role in the Panx3 ER Ca^2+^ channel function. We analyzed Ca^2+^ efflux from the ER and stimulation of Ca^2+^ storage by ATP. The Ser68Ala mutation inhibited Panx3-mediated Ca^2+^ efflux (Fig. 5A). The other functions of Panx3, such as ATP release from the hemichannel and Ca^2+^ propagation of gap junction activity, were not affected by Ser68Ala (Fig. 5B,C and Supplementary Movie 1–3). Thus, P-Panx3 affects only the ER Ca^2+^ channel function among the three Panx3 channel functions.

**Figure 6:**
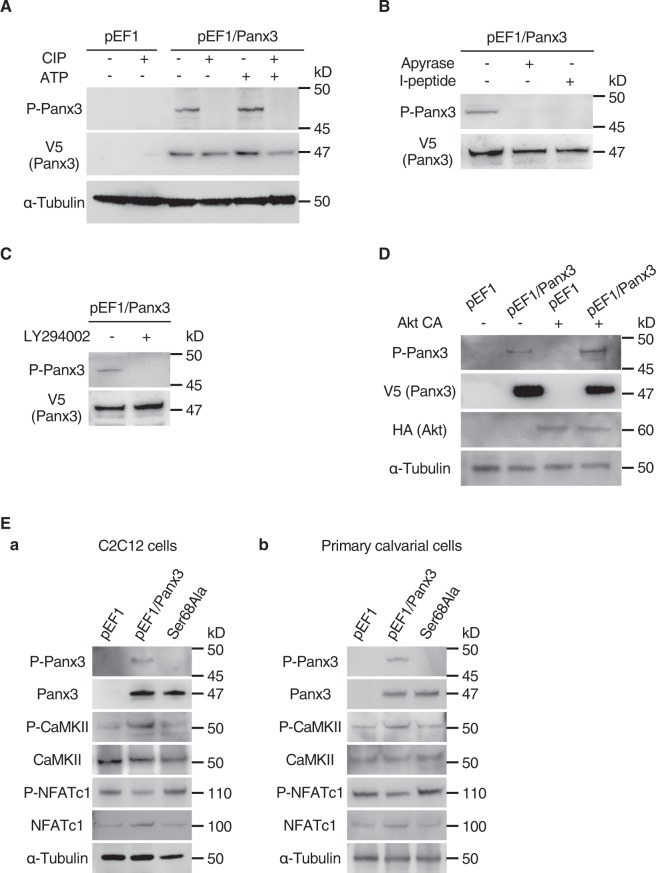
ATP/PI3K/Akt signaling phosphorylates Panx3 at Ser68. (**A**) Western blotting with antibodies to P-Panx3, V5 (Panx3), and α-tubulin with or without CIP treatment and ATP stimulation in C2C12 cells stably transfected with pEF1 or pEF1/Panx3. Proteins were isolated after 30 sec of ATP stimulation and treated with CIP (80 U) before loading. (**B**) Cells stably transfected with pEF1/Panx3 were cultured for 1 h with 20 U Apyrase (ATP receptor antagonist) or Panx3 hemichannel inhibitory peptide (I-peptide; 10 μg/ml) before protein isolation. (**C**) Cells stably transfected with pEF1/Panx3 were cultured for 30 min with 100 μM LY294002 (PI3K inhibitor) before protein isolation. (**D**) Cells stably transfected with pEF1 and pEF1/Panx3 were transfected with Akt CA or mock vector and cultured in DMEM for 1 day. Western blotting was conducted with HA antibody to determine exogenous Akt expression. (**E**) Ser68Ala inhibited Panx3-activated CaM/NFATc1 signaling pathways. C2C12 cells (a) or primary calvarial cells (b) were stably and transiently transfected with pEF1, pEF1/Panx3, or Ser68Ala vectors, and incubated for 1 h with BMP2, and the levels of the Ca^2+^ signal molecules P-CaMKII, CaMKII, P-NFATc1, and NFATc1 were analyzed by western blotting. Western blot was perfomed by at least three independent experiments. Full blot is shown in the Supplemental Information (Full Original Blots-IV).

We next examined the regulatory mechanisms of Panx3 ER Ca^2+^ channel gating by P-Panx3. ATP stimulated the Panx3 ER Ca^2+^ channel (Fig. 5A), and since we previously reported that the Panx3 ER Ca^2+^ channel is regulated by the ATP/PI3K/Akt signaling pathway^5^, we used western blotting to analyze P-Panx3 expression following ATP stimulation. ATP stimulation increased P-Panx3 expression, and this was reversed by CIP treatment and use of the ATP inhibitor, apyrase (Fig. 6A,B). Addition of the Panx3 hemichannel inhibitory peptide (I-peptide) also diminished P-Panx3 expression (Fig. 6B). The PI3K inhibitor, LY294002 also inhibited P-Panx3 (Fig. 6C). The PI3K downstream molecule, Akt constitutive active vector (Akt CA), increased P-Panx3 expression (Fig. 6D). These results suggest that the ATP/PI3K/Akt signaling pathway regulates P-Panx3.

We also examined the Ca^2+^ signaling pathway in C2C12 cells and in primary calvarial cells transfected with a Ser68Ala vector and induced by BMP2 for 1 h. Ser68Ala inhibited the Panx3-mediated Ca^2+^ signaling pathway (Fig. 6E,a,b).

**Figure 7:**
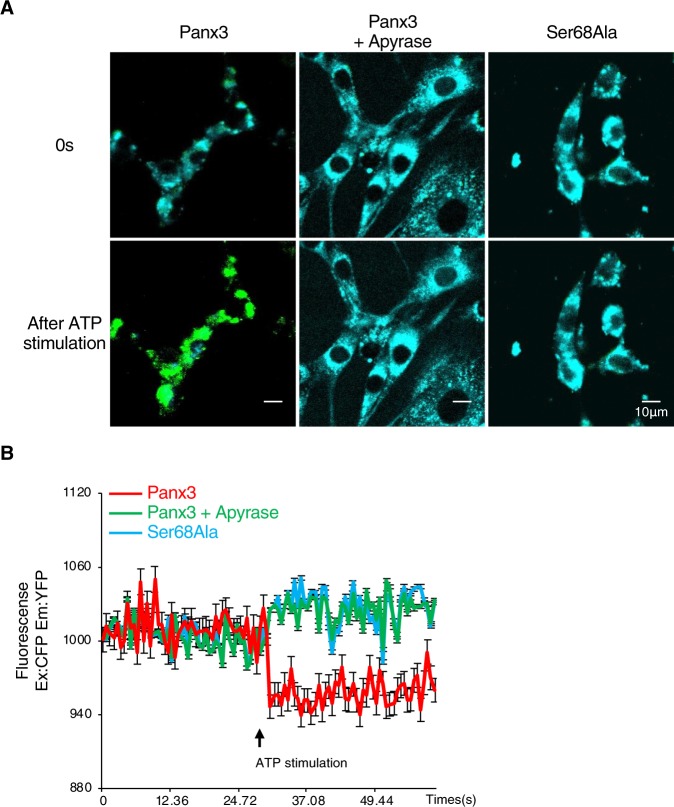
Ser68Ala inhibits changing of Panx3 protein conformation after ATP stimulation. (**A**) Real-time FRET images represent the CFP/YFP emission ratio of Panx3 protein conformation in cells transiently co-transfected with CFP-Panx3 vector and Panx3-YFP vector with or without apyrase or in cells transiently co-transfected with CFP-Ser68Ala vector and Ser68Ala-YFP vector and treated with ATP (n = 5). Signals were excited by 458 nm Argon laser and detected between 468–509 nm (CFP, donor fluorescence: cyan) and 519–620 nm (YFP, acceptor fluorescence: green). (**B**) The time courses represent the FRET ratio by ATP stimulation in Panx3-transfected and Ser68Ala-transfected cells with or without apyrase. Average traces of five different experiments are shown with the solid lines. The arrow indicate the time of ATP stimulation.

We then used fluorescence resonance energy transfer (FRET) analysis to examine the changes in Panx3 protein conformation by phosphorylation to P-Panx3. We created recombinant Panx3 or Ser68Ala with CFP to the N-terminus and YFP to the C-terminus. The FRET real-time imaging of cells co-transfected with CFP and YFP vectors showed that Panx3-expressing C2C12 cells showed FRET after ATP stimulation, while cells treated with apyrase or Ser68Ala expressing cells did not (Fig. 7A and Supplementary Movies 4–6). Quantification of FRET with a luminometer also generated similar results as imaging (Fig. 7B). The FRET analyses indicate that the protein conformation of the Panx3 channel was transformed by ATP-induced phosphorylation at Ser68 to control Panx3 ER Ca^2+^ channel gating.

## Discussion

**Figure 8:**
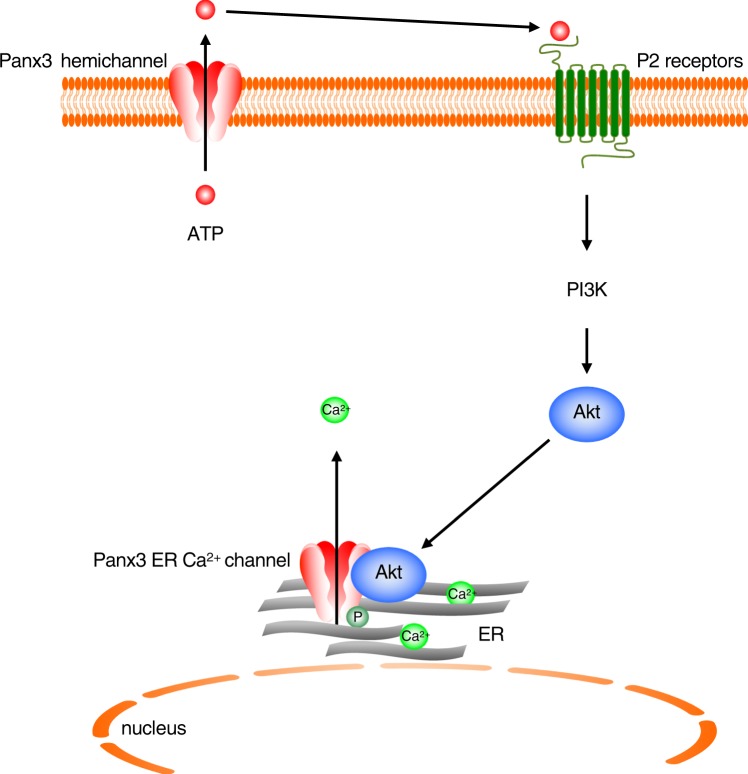
The mechanism of Panx3 ER Ca^2+^ channel gating by Akt mediated phosphorylation at Ser68. The Panx3 hemichannel releases intracellular ATP to extracellular spaces. Extracellular ATP binds P2 receptors in an autocrine or paracrine manner, which promotes Akt activation though PI3K. Akt then activates Panx3 ER Ca^2+^ channel to promote Panx3 ER Ca^2+^ channel activity.

In this study, we demonstrated that the Panx3 ER Ca^2+^ channel is regulated by ATP/PI3K/Akt mediated phosphorylation at the Ser68 residue. P-Panx3 has a pronounced involvement in osteoblast differentiation but not in osteoprogenitor proliferation. P-Panx3 is localized on the ER membrane, and mutation of Ser68 inhibits Ca^2+^ efflux from Panx3 ER Ca^2+^ channel, but does not affect the ATP releasing hemichannel or the gap junction function, with the end result of propagation of intracellular Ca^2+^ to neighboring cells. The Panx3 hemichannel releases intracellular ATP to the extracellular space to promote the activation of the PI3K/Akt signaling pathway through purinergic receptors. Panx3 then undergoes phosphorylation at Ser68 on the ER membrane (Fig. 8). The resulting P-Panx3 changes the Panx3 protein conformation to release Ca^2+^ from ER. These results indicate that the gating of the Panx3 ER Ca^2+^ channel is regulated by phosphorylation and the gating of each of the three types of Panx3 channels is regulated by different mechanisms.

The Panx3 ER Ca^2+^ channel is opened by phosphorylation at the Ser68 residue. For the phosphorylation of Panx3 at Ser68, it is possible that Panx3 forms a protein complex with Akt (Fig. 6D). Global *Akt-1* KO mice have bone abnormalities that are evident as a reduction in bone mineral density^32^. The global *Akt-2* KO mice develop severe diabetes, and yet they display only a small reduction in weight and length^33^. *Akt-1/Akt-2* double KO mice die shortly after birth and exhibit dwarfism^34^, while *Akt3* KO mice are viable and have reduced brain size, but no apparent mutant skeletal phenotype^35^. The *Panx3* KO mice revealed severe dwarfism and reduction in bone density^26^. Comparison of the mouse phenotypes between *Panx3* KO mice and *Akt* KO mice suggested that the Panx3 ER Ca^2+^ channel may be regulated by Akt-1. We still remain unclear how Ser68 of Panx3 at ER membrane can be phosphorylated by a cytoplasmic kinase, Akt. We also were unable to ascertain whether Akt binds Panx3 directly or indirectly. The sequence of the Ser68 site is not the binding consensus sequence for Akt^36,37^. Thus, one possibility is that the protein complex formed with Akt and other molecules and signaling pathway controlled by them may require to activate the Panx3 ER Ca^2+^ channel. We need further experiments to answer the question.

Several kinases are reported to bind and activate Akt^38–40^. One of these, 5′ AMP-activated protein kinase (AMPK) may form a complex with Akt to activate the Panx3 ER Ca^2+^ channel. We recently found that Panx3 regulates proliferation and differentiation of odontoblasts by AMPK activity through ATP releasing hemichannel activities^17^. *AMPKα1* and *AMPKα2* KO mice are viable, but *AMPKα1/α2* double KO mice show embryonic lethality^41^. The *AMPKα1* KO mice have severely low bone mass, with both cortical and trabecular bone compartments being smaller^42,43^. Thus, AMPK could be involved in Panx3 channel regulation. Another potential candidate is BMP2 signaling, because it regulates Panx3 expression and activation of the Panx3 signaling pathway^5,25^. Thus, BMP2 may contribute to the activation of Panx3 channel. In present study, BMP2 was added for cell culture conditioning in several differentiation experiments. The results suggest that BMP2 may regulate Panx3 channel activation by phosphorylation. BMP2 signaling has two main pathways—smad dependent or independent^44^. Thus, smad-dependent signaling and an independent pathway, such as MAPK signaling, may interact to cause Panx3 ER Ca^2+^ channel activation.

Candidate phosphorylation sites other than Ser68 may also exist in Panx3 and may contribute to the regulation of gating of the Panx3 channel, hemichannel, and gap junction by other mechansims. The hemichannel and gap junction are located on the plasma membrane. The gap junction forms between adjacent cells, whereas the hemichannel forms from the cell to the extracellular space. We can therefore expect that the activation mechanisms for each of the hemichannel, gap junction, and ER Ca^2+^ channel types would differ. In our screening with the ALP assay, we found another interesting site, Ser303 (Fig. 2B). Mutation of Ser303 to Ser303Ala also inhibited Panx3 mediated ALP activity, although the inhibition ratio for Ser303Ala was less than that for Ser68Ala. Ser68Ala inhibited Panx3-mediated osteoblast differentiation, but not proliferation, and inhibited Panx3 ER Ca^2+^ channel activity (Fig. 2D–F), whereas Ser303Ala inhibited Panx3-mediated osteoblast differentiation and osteoprogenitor cell proliferation (unpublished data). Panx3 inhibits osteoprogenitor cell proliferation by regulating Wnt and p21 signaling^25^. The inhibition of cell proliferation by Panx3 is regulated by the Panx3 hemichannel activity and the Panx3 hemichannel is the first action channel among the three Panx3 channels. Since Ser303Ala affected cell proliferation, phosphorylation of Ser303 may contribute to Panx3 hemichannel activity strongly. We need further analysis to address this hypothesis.

In terms of gap junction activity, we have not yet identified a candidate phosphorylation site for its control. The directions of the extracellular and intracellular domains of the gap junction should be the same as those of the hemichannel because they are localized on the plasma membrane. However, since gap junction proteins connect to one another inside neighboring cells to form the gap junction, the gating mechanism of gap junction may have different actions from the others. Panxs are also recognized as glycosylation proteins^28,29^, and glycosylation regulates the trafficking of the Panxs protein to the cell surface and its docking with an adjacent Panxs channel in a neighboring cell to form a gap junction^45^. Overexpression of N-glycosylation deficient mutants of Panx1 and Panx3 in HeLa cells prevented dye transfer between the cells^19^. This finding suggests that glycosylation may regulate the contraction of the gap junction. However, the mechanisms controlling Panx3 gap junction activation are still unclear. We cannot ignore the possibility that phosphorylation also may be involved in gap junction gating. Confirmation of this possibility will require further analysis. Our findings suggest that the phosphorylation of Ser68 activates only the Panx3 ER Ca^2+^ channel activity, not the hemichannel and gap junction activities. These results and the differences in location between each Panx3 channel clearly indicate that each of the three Panx3 channels have their own regulation mechanisms for gating.

Each of the three Panx3 functions orchestrate and harmonize bone formation. Panx3 is induced at the transition stage from cell proliferation to differentiation. Its expression is first induced at the early osteoblast differentiation stage, and it decreases at the mature osteoblast differentiation stage^26^. The Panx3 expression pattern does not indicate how each channel activates and contributes to the other. In this study, the Panx3 ER Ca^2+^ channel was phosphorylated by extracellular ATP released from the Panx3 hemichannel following the activation of PI3K/Akt signaling. In the growth plate, P-Panx3 was expressed in the prehypertrophic, hypertrophic zone, and bone areas. The expression level of P-Panx3 was higher in prehypertrophic chondrocytes than in hypertrophic ones, when compared with normal Panx3 expression in the growth plate (Fig. 3D). Most of the P-Panx3 positive cells were not merged with Ocn expressing cells in the bone area, nor was the P-Panx3 expression pattern completely the same as the Panx3 expression pattern. These results suggest that P-Panx3 occurs at an early stage and its level is reduced in the mature stage during development. The transition of cells from a proliferative to a differentiation state would be expected to require a substantial energy flow and huge exchanges of energy. The control of intracellular Ca^2+^ levels by the Panx3 ER Ca^2+^ channel predicts that phosphorylation would happen mainly at the transition stage.

The gap junction proteins that are known to regulate bone formation are Panx3 and Cx43. These two proteins have their own distinct functions and expression patterns that function in their contribution to bone growth. Panx3 functions as an ER Ca^2+^ channel to promote differentiation, and it can rescue mineralization defects in Cx43^−/−^ calvarial cells^26^, while Cx43 is a phosphorylated gap junction protein^46^. The cytoplasmic C-terminal domain of Cx43 is important for the formation of gap junction channels^46^, and its C-terminal domain contains 32 amino acid residues that are phosphorylated by protein kinases^47,48^, with 15 serine residues phosphorylated by Ca^2+^/calmodulin protein kinase II (CaMKII)^49^. Cx43 is also well known as a phospho-substrate for mitogen-activated protein (MAP) kinases, as well as protein kinase C (PKC)^50–52^. The phosphorylation of Cx43 at the S368 residue reduces hemichannel activity^53^. Cx43 is also reported to be phosphorylated by Akt to traffic and stabilize the Cx43 protein in the plasma membrane^54^. Our present findings would allow the addition of a distinct regulation mechanism between Panx3 and Cx43. Although many molecules, including kinases, activate Cx43 channels, Akt works in different systems to control Panx3 and Cx43 channel gating.

In summary, we have shown that the Panx3 ER Ca^2+^ channel was phosphorylated at Ser68 residue by ATP/PI3K/Akt signaling. This phosphorylation mechanism is critical for promoting osteoblast differentiation. Our results reveal that the three Panx3 channels each have different activation mechanisms and function together to orchestrate osteogenesis.

## Materials and Methods

### Reagents

A rabbit polyclonal P-Panx3 antibody was raised against a synthetic peptide (amino acid residues, SCFpSPSNFSC) from the first extracellular loop of the mouse Panx3 protein and the native peptide was used as a control sequence (SCFSPSNFSC). Rabbit anti-Panx3 antibody was used as previously described^5,16^. The Panx3 expression vector (pEF1/Panx3) and the control vector (pEF1) have been described previously^16^. In brief, the pEF1/Panx3 vector was constructed by cloning the coding sequence of mouse Panx3 cDNA into the pEF1/V5-His vector (Invitrogen). The antibodies were obtained for Ocn from Biomedical Technology; CaMKII and P-CaMKII from Cell Signaling Technology; P-NFATc1 from Santa Cruz Biotechnology, Inc.; V5 from Invitrogen; and NFATc1 from BD. HA was from COVANCE; α-tubulin from Sigma-Aldrich, CIP from New England Biolabs; Apyrase from Sigma-Aldrich; LY294002 from Invitrogen; BMP2 from Humanzyme; and iQ SYBR Green Supermix from Bio-Rad Laboratories. ER-tracker was obtained from Invitrogen. HRP-conjugated goat anti–mouse and goat anti–rabbit IgG were obtained from United States Biological. The Akt-CA was obtained from Addgene. The inhibitory Panx3 peptide was described previously^16^.

### Cell culture

C2C12 cells were grown in DMEM (Invitrogen) containing 10% FBS (HyClone) at 37 °C under 5% CO_2_. For the experiments, C2C12 cells were induced from osteogenic cells by the addition of 50 µg/ml ascorbic acid (Sigma-Aldrich) and 5 mM β-glycerophosphate (Sigma-Aldrich). For the proliferation assay, the cells (2.5 × 10^3^ cells/ml) were stably transfected with either pEF1/Panx3 or Ser68Ala or control pEF1 and cultured in 96-well plates for 2 days. The cell proliferation activity was evaluated using a cell counting kit (Dojindo). The absorbance was measured using a microplate reader. For osteoblast differentiation, transiently or stably transfected C2C12 cells (∼90% confluence) were cultured in the presence of 300 ng/ml BMP2 (Humanzyme) and 2% FBS. Primary calvarial cells were prepared from the calvaria of newborn mice and cultured in α-minimum essential medium (α-MEM; Invitrogen) with 10% FBS, 100 units/ml of penicillin, and 100 µg/ml of streptomycin, as previously described^55^. For the experiment with primary calvarial cells, cells were induced by osteogenesis with 50 µg/ml ascorbic acid (Sigma-Aldrich) and 5 mM β-glycerophosphate (Sigma-Aldrich). All experimental procedures were approved by the Animal Care and Use Committee of the National Institute of Dental and Craniofacial Research (protocol no. 15-758) and Tohoku University (protocol no. 2017DnA-035). And all methods were carried out in accordance with relevant laws and legislations of both NIDCR and Tohoku Univeristy.

### *In vitro* phosphatase assay

Protein lysates isolated using PhosphoSafe extraction buffer (EMD Millipore) were run on SDS-PAGE. The phosphorylation band was analyzed by staining with Pro-Q Diamond phosphoprotein gel stain kit (Invitrogen) following the company’s standard protocol. Briefly, immunoprecipitated cell lysate by V5 antibody was loaded into NuPAGE Bis-Tris gels (Thermo). The gel was immersed in a fixed solution (50% methanol and 10% acetic acid) and then incubated at room temperature with gentle shaking for 1 hour. After washing, the gel was stained with Pro-Q Diamond phosphoprotein gel stain, with gentle agitation in the dark for 90 minutes. To reduce the background, the gel then was destained by incubating the gel in a destaining solution (Thermo) with gentle shaking for 30 minutes at room temperature, protected from light. Detaining was repeated several times until the background was reduced.

### Enzyme-linked immunosorbent assay (ELISA) and multiplex ELISA

Cytokines were measured by conventional ELISA (R&D Systems, BD, and elisakit.com) or multiplex ELISA (Quansys Biosciences). Both methods were performed as recommended by the manufacturer. If appropriate, cytokine data were normalized to total protein data (Pierce BCA Protein Assay Kit; Thermo Scientific) with the following formula: raw cytokine data (in pg/ml) / total protein concentration in the lysate (in mg/ml).

### ALP assay

The phosphorylation sites of interest in Panx3 were screened by constructing a mutation vector of each of 17 phosphorylation candidate residues using a QuikChange XL Site-Directed Mutagenesis Kit (Agilent Technologies). Each mutation construct was transiently transfected into C2C12 cells with Nucleofector (Lonza). The transfected cells were then plated into 96-well culture plates and grown to 100% confluence. The cells were then induced to differentiate into osteoblasts by addition of 300 ng/ml BMP2. The ALP activity was measured in cell layers using a p-nitrophenyl phosphate substrate and an incubation temperature of 37 °C, or it was determined by the tartrate-resistant acid phosphatase (TRACP) & ALP double-stain kit (Takara Bio Inc.). The protein concentration was determined by the BCA protein assay method (Thermo Fisher Scientific).

### Immunostaining

For immunostaining of newborn growth plates, the tissues were fixed overnight in paraformaldehyde, embedded in paraffin, and cut into 10-µm sections. After deparaffinization and rehydration, the sections were processed for heat-induced epitope retrieval in pH 6.0 citrate buffer (Dako). The cultured cells were stained by first incubating them in ER-Tracker Red (Invitrogen) for 20 min, followed by fixation in acetone at −20 °C for 2 min. The cells were blocked with Power block (Biocare Medical) and reacted for 2 h at room temperature with primary antibodies. The primary antibodies were detected by Alexa 488 (Invitrogen) or Alexa 594 (Invitrogen) or by Cy-5–conjugated (Jackson ImmunoResearch Laboratories) secondary antibodies. Nuclear staining was performed using Hoechst dye (Sigma-Aldrich). The analysis was performed on an LSM 780 inverted confocal microscope (Carl Zeiss MicroImaging, Inc.).

### Immunoelectron microscopy

For immunolocalization of P-Panx3 protein, glutaraldehyde-fixed, Lowicryl-embedded thin sections were first reacted with the primary antibody diluted in PBS with 1% BSA and 0.1% Triton X-100 (1/100 diluted anti-P-Panx3) for 1 h at room temperature. After washing in PBS, grids were incubated for 30 min at room temperature with the goat 1/25 diluted anti-rabbit secondary antibody (Nanoprobes) coupled to 10 nm gold particles diluted in PBS. Following final washing in PBS, the grids were rapidly rinsed in a jet of distilled water, air-dried, and counterstained with 4% uranyl acetate. The sections were analyzed with a Hitachi H7600 transmission electron microscope.

### ATP flux

The ATP flux was examined by luminometry, as previously described^5,16^. C2C12 cells stably expressing pEF1, pEF1/Panx3, or Ser68Ala were seeded at 1.0 × 10^4^ cells/well in a 96-well plate, and cultured for 2 days in DMEM containing 10% FBS. The cells were then washed with PBS, followed by incubation in PBS for 2 min. The supernatant was collected and assayed with luciferase and luciferin (Promega). The luminescence was measured using a Mithras LB 940 multimode plate reader (Berthold).

### Measurement of [Ca^2+^]_i_ and imaging of Ca^2+^ wave propagation

The [Ca^2+^]_i_ and imaging of Ca^2+^ wave propagation were examined as previously described^5^. The [Ca^2+^]_i_ measurements were conducted on C2C12 cells stably expressing pEF1, pEF1/Panx3, or Ser68Ala cultured in a 96-well plate for 3 days cultured with DMEM containing 10% FBS and then loaded with 5 µM Fura-2AM (Invitrogen) in Ca^2+^ containing HBSS for 45 min at 37 °C in 5% CO_2_. The Ca^2+^ transients in Ca^2+^-free HBSS were recorded as the 340/380 nm ratio (R) of the resulting 510 nm emissions determined using a plate reader (TriStar^2^ LB942; Berthold Technologies). For the stimulation, 200 µM of ATP was automatically injected into the cell cultures by the LB942. The Ca^2+^ wave propagation was imaged by seeding C2C12 cells stably expressing pEF1, pEF1/Pnax3, or Ser68Ala in a glass-bottomed dish and incubating in Ca^2+^ containing HBSS containing 4 µM Fluo-4 AM Ca^2+^ indicator (Invitrogen), 10 µM pluronic F-127 (Invitrogen), 0.1% OxyFluor (Oxyrase), and 2.5 µM caged reagent NP-EGTA AM (Invitrogen) for 30 min at room temperature, followed by washing and incubation with Ca^2+^-free HBSS. The Ca^2+^ was uncaged by a two-photon laser set at 730 nm focused on a single cell. The Ca^2+^ wave propagation was measured at 10 s after the illumination.

### Time-lapse FRET imaging and measurement

The plasmids encoding FRET biosensors were constructed by cloning the coding sequence of mouse normal or Ser68 mutated Panx3 cDNA into the pEYFP-N1 and pECFP-C1 vectors (Clontech). C2C12 cells transfected with biosensors were plated on glass-bottomed dishes and cultured with Phenol-Red-free DMEM containing 10% FBS. Time-lapse FRET imaging was performed on a Zeiss LSM 780 confocal microscope (Carl Zeiss MicroImaging, Inc.). The donor and transfer signals were excited by the 458 nm line of an Argon laser and detected simultaneously between 463–509 nm and 519–620 nm. Images were collected from the middle plane of cells. Time-lapse FRET imaging was captured for 30 s after ATP stimulation. To inhibit ATP receptors, the cells were incubated with 20|U Apyrase before capturing the images. To measure FRET ratio, FRET biosensor vectors including normal or Ser68 mutated Panx3 cDNA transiently transfected C2C12 cells were cultured in a 96-well for 2 days. The FRET ratio was recorded as the 485/536 nm ratio using a plate reader (TriStar^2^ LB942; Berthold Technologies). 200 µM of ATP stimulation was auto-injected by the LB942.

### Western blot analysis

The cell lysates were prepared as previously described^16^. A 10 µg sample of each protein was electrophoresed in 4–12% SDS-polyacrylamide gel (Invitrogen) and transferred onto a polyvinylidene difluoride membrane using an iBlot device (Invitrogen). The membranes were immunoblotted with antibodies using standard protocols.

### Data analysis

Each experiment was repeated several times and the data were analyzed using Prism 5 software. Student’s t-tests were used to highlight differences between two groups of data. One-way ANOVA was used for the quantification of band density ratios (Fig. 1A,b), ALP activity (Fig. 2B.F), cell proliferation (Fig. 2D), qPCR data (Fig. 2E), colocalization (Figs. 4A,b and S1B). P < 0.05 was considered statistically significant.

## Supplementary information

Supplementary information

Supplemental Movie 1

Supplemental Movie 2

Supplemental Movie 3

Supplemental Movie 4

Supplemental Movie 5

Supplemental Movie 6
