# Supplementary material for: Pannexin 3 ER Ca2+ channel gating is regulated by phosphorylation at the Serine 68 residue in osteoblast differentiation
Source: Sci Rep. 2019 Dec 10;9:18759. doi: 10.1038/s41598-019-55371-9 (PMC6904572; doi:10.1038/s41598-019-55371-9)
Supplement: Supplementary file 1 — Supplementary information [file 41598_2019_55371_MOESM1_ESM.pdf]

Supplementary Information

Pannexin 3 ER Ca^{2+} channel gating is regulated by phosphorylation at the Serine 68 residue in osteoblast differentiation.

Masaki Ishikawa[1]‡, Geneva Williams[2], Patricia Forcinito[2], Momoko Ishikawa[3], Ryan J. Petrie[4], Kan Saito[3], Satoshi Fukumoto[3], and Yoshihiko Yamada[2]

[1]Operative Dentistry, Tohoku University Graduate School of Dentistry, Sendai 980-8575, Japan

[2]Molecular Biology Section, National Institute of Dental and Craniofacial Research, National Institutes of Health,

[3]Department of Pediatric Dentistry, Tohoku University Graduate School of Dentistry, Sendai 980-8576, Japan

[4]Department of Biology, Drexel University, Philadelphia, PA 19104, USA

‡Address correspondence to: Masaki Ishikawa, Department of Restorative Dentistry, Division of Operative Dentistry, Tohoku University, Graduate School of Dentistry 4-1, Seiryo chou, Aoba-ku, Sendai, Miyagi, 980-8575, Sendai, Japan. Tel: 011-81-22-717-8343; Fax: 011-81-22-717-8344; E-mail: ishikawamas@dent.tohoku.ac.jp.

SUPPLEMENTARY INFORMATION LEGENDS

Supplementary Figure 1. (A) Cellular localization of cell stably co-transfected with CFP-Panx3 and Panx3-YFP vector (upper) and CFP-Ser68Ala and Ser68-YFP vector (bottom). Fluorescent confocal images showed CFP-Panx3 or Ser68Ala (left, cyan), Panx3 or Ser68Ala-YFP (middle, green) and merged (right, light green). (B) Measurements show a percentage of colocalization between CFP with YFP. NS, nonsignificant.

Supplementary Movie 1. Real-time Ca^{2+} wave propagation in C2C12 cells transfected with control pEF1 vector. The Ca^{2+} wave was measured in C2C12 cells stably transfected with the control pEF1 vector, then loaded with Fluo-4 and NP-EGTA (caged Ca^{2+}) by initiating photolysis of NP-EGTA in a single cell using laser illumination.

Supplementary Movie 2. Real-time Ca^{2+} wave propagation in C2C12 cells transfected with pEF1/Panx3 vector. The Ca^{2+} wave was measured in C2C12 cells stably transfected with the pEF1/Panx3 vector, then loaded with Fluo-4 and NP-EGTA (caged Ca^{2+}) by initiating photolysis of NP-EGTA in a single cell using laser illumination.

Supplementary Movie 3. Real-time Ca^{2+} wave propagation in C2C12 cells transfected with Ser68Ala vector. The Ca^{2+} wave was measured in C2C12 cells stably transfected with the Ser68Ala vector, then loaded with Fluo-4 and NP-EGTA (caged Ca^{2+}) by initiating photolysis of NP-EGTA in a single cell using laser illumination.

Supplementary Movie 4. Real-time FRET images represent the CFP/YFP emission ratio of Panx3 protein conformation in cells transiently co-transfected with CFP-Panx3 vector and Panx3-YFP vector without apyrase. Time-lapse FRET imaging was captured for 30 s after ATP stimulation.

Supplementary Movie 5. Real-time FRET images represent the CFP/YFP emission ratio of Panx3 protein conformation in cells transiently co-transfected with CFP-Panx3 vector and Panx3-YFP vector with apyrase (20 U). Time-lapse FRET imaging was captured for 30 s after ATP stimulation.

Supplementary Movie 6. Real-time FRET images represent the CFP/YFP emission ratio of Panx3 protein conformation in cells transiently co-transfected with CFP-Ser68Ala vector and Ser68Ala-YFP vector without apyrase. Time-lapse FRET imaging was captured for 30 s after ATP stimulation.

Full Original Blots-I.

Panel Aa: These figures display the full original blots for Fig.1Aa shown in the text/Results. Panel Ba: These figures display the full original blots for Fig.1Ba shown in the text/Results. The identification of bands was based on the expected molecular weight. For details see legend of Fig.1. The same blot of V5 antibody was stripped and re-blotted using anti-α-tubulin antibody for loading control. Areas presented in the manuscript figures are boxed.

Full Original Blots-II.

Panel C: These figures display the full original blots for Fig.2C shown in the text/Results. The identification of bands was based on the expected molecular weight. For details see legend of Fig.2. The same blot of V5 antibody was stripped and re-blotted using anti-α-tubulin antibody for loading control. Areas presented in the manuscript figures are boxed.

Full Original Blots-III.

Panel B: These figures display the full original blots for Fig.3B shown in the text/Results. Panel C: These figures display the full original blots for Fig.3C shown in the text/Results. The identification of bands was based on the expected molecular weight. For details see legend of Fig.3. The same blot of V5 (Panel B) or Panx3 (Panel C) antibody was stripped and re-blotted using anti-α-tubulin antibody for loading control. Areas presented in the manuscript figures are boxed.

Full Original Blots-IV.

Panel A: These figures display the full original blots for Fig.6A shown in the text/Results. Panel B: These figures display the full original blots for Fig.6B shown in the text/Results. Panel C: These figures display the full original blots for Fig.6C shown in the text/Results. Panel D: These figures display the full original blots for Fig.6D shown in the text/Results. Panel Ea and b: These figures display the full original blots for Fig.6Ea and b shown in the text/Results. The identification of bands was based on the expected molecular weight. For details see legend of Fig.6. The same blot of Panx3 antibody in Panel E was cut at around 60 kDa, stripped and re-blotted using anti-α-tubulin antibody for loading control. Areas presented in the manuscript figures are boxed.

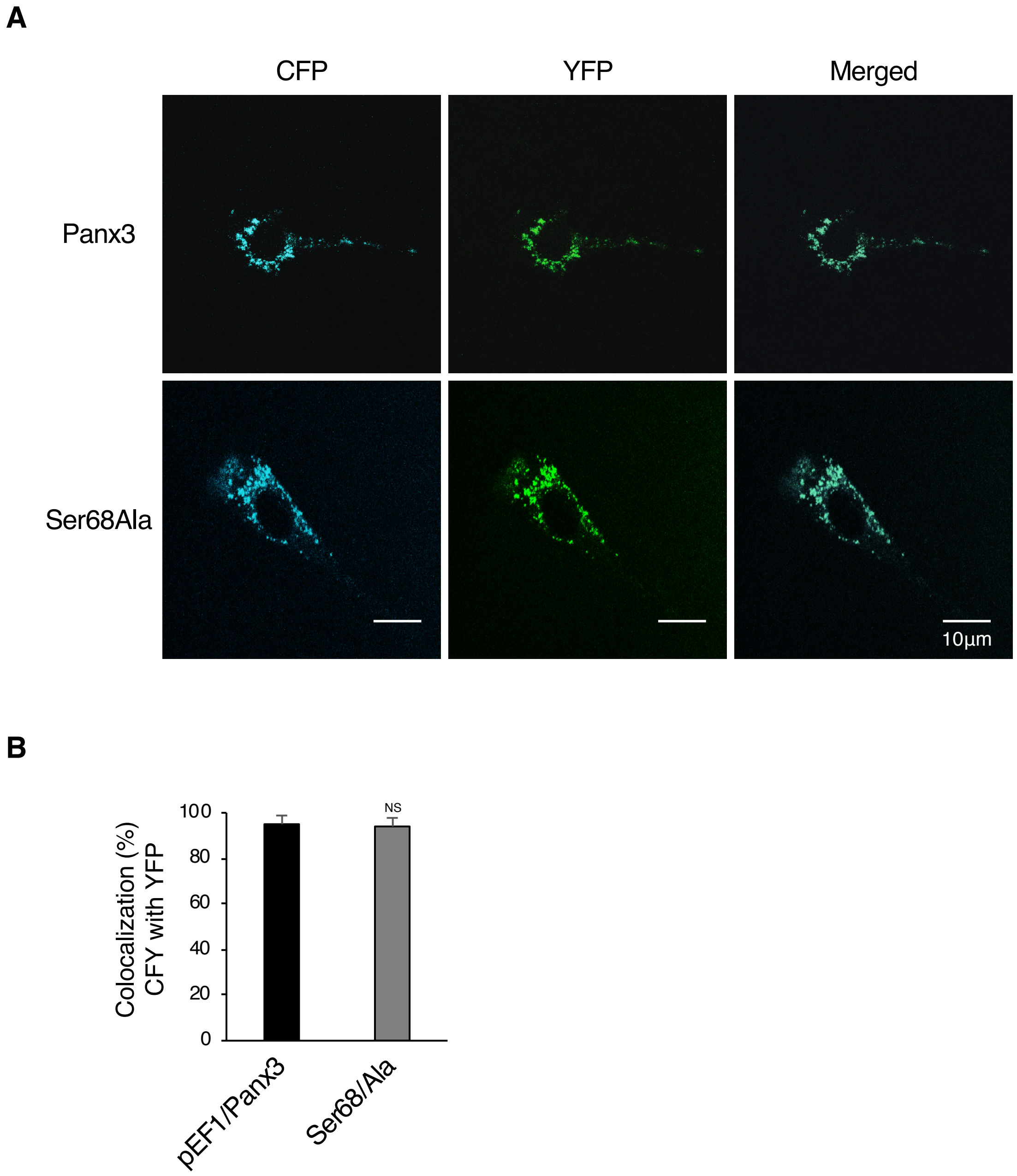
A
CFP
YFP
Merged
Panx3
Ser68Ala
10μm
B
Colocalization (%)
CFY with YFP
100
80
60
40
20
0
NS
pEF1/Panx3
Ser68/Ala

A
a

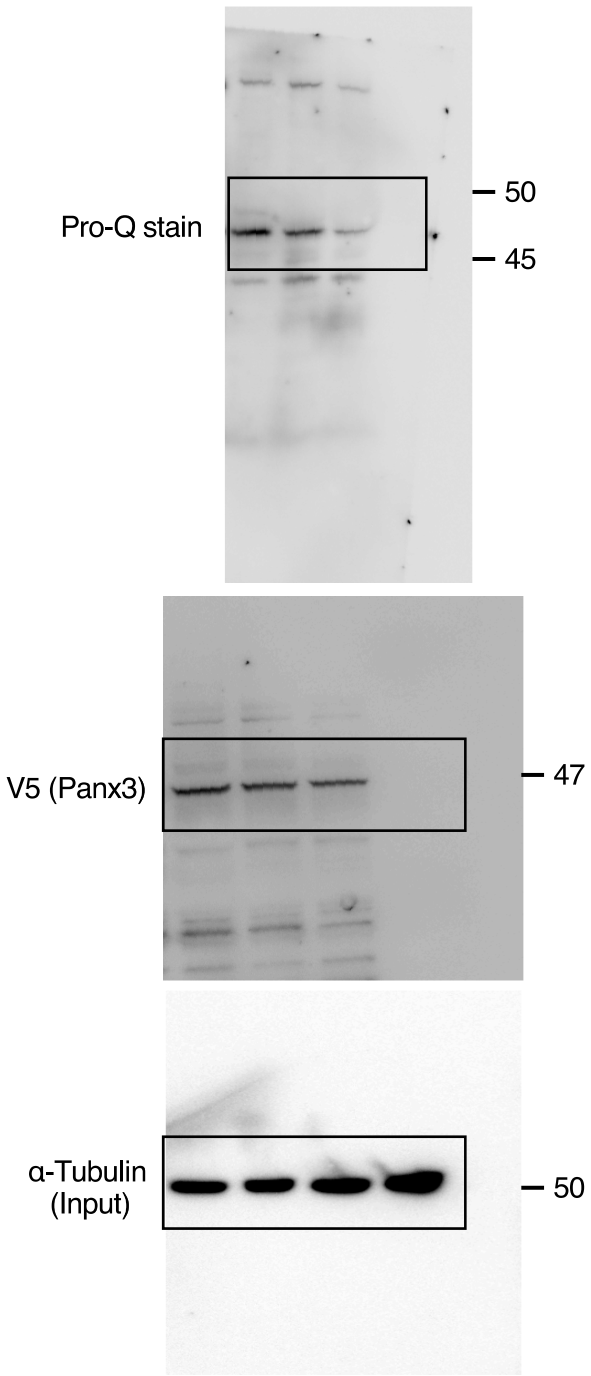
Pro-Q stain
50
45
V5 (Panx3)
47
α-Tubulin
(Input)
50

B
a

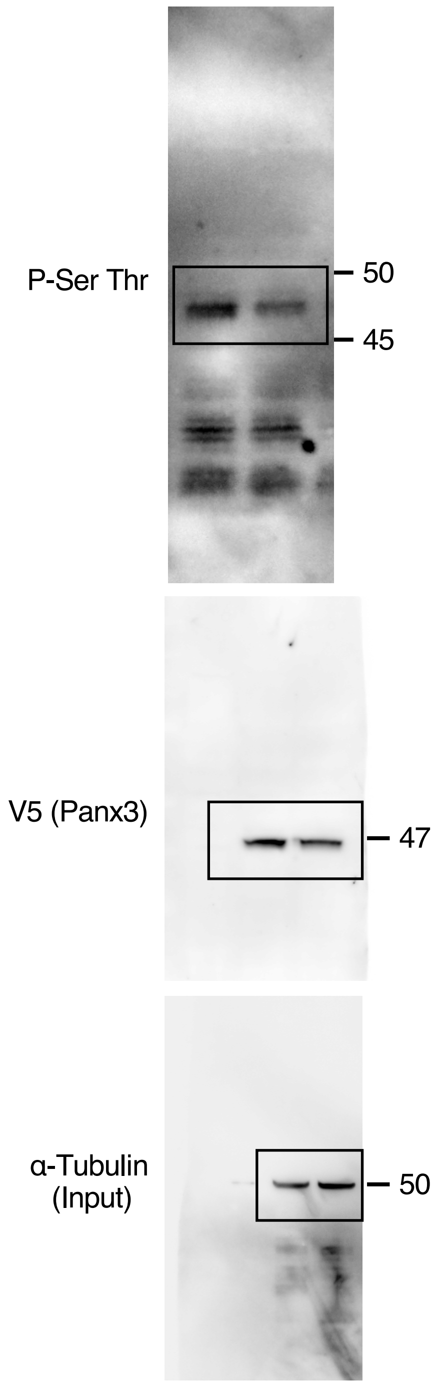
P-Ser Thr
50
45
V5 (Panx3)
47
α-Tubulin
(Input)
50

II

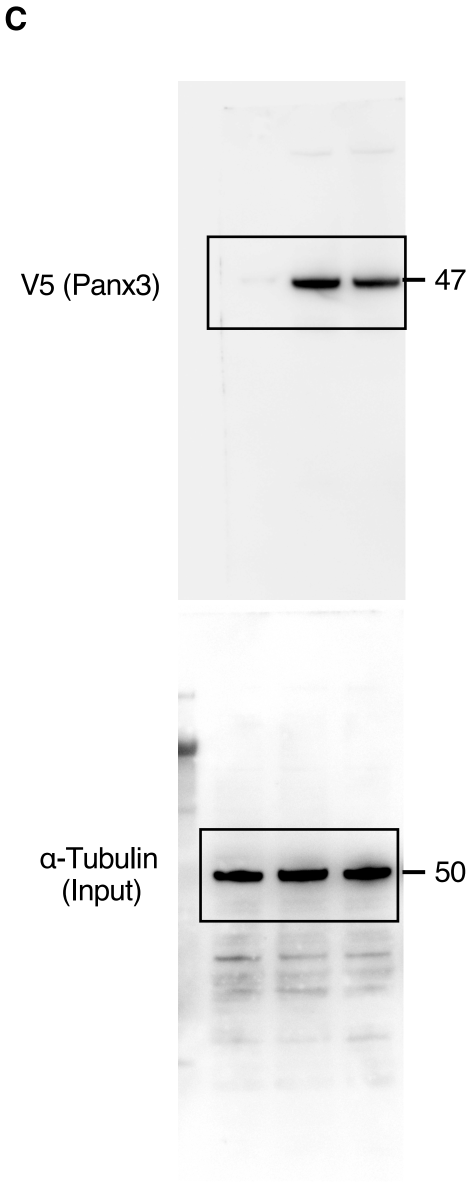

III

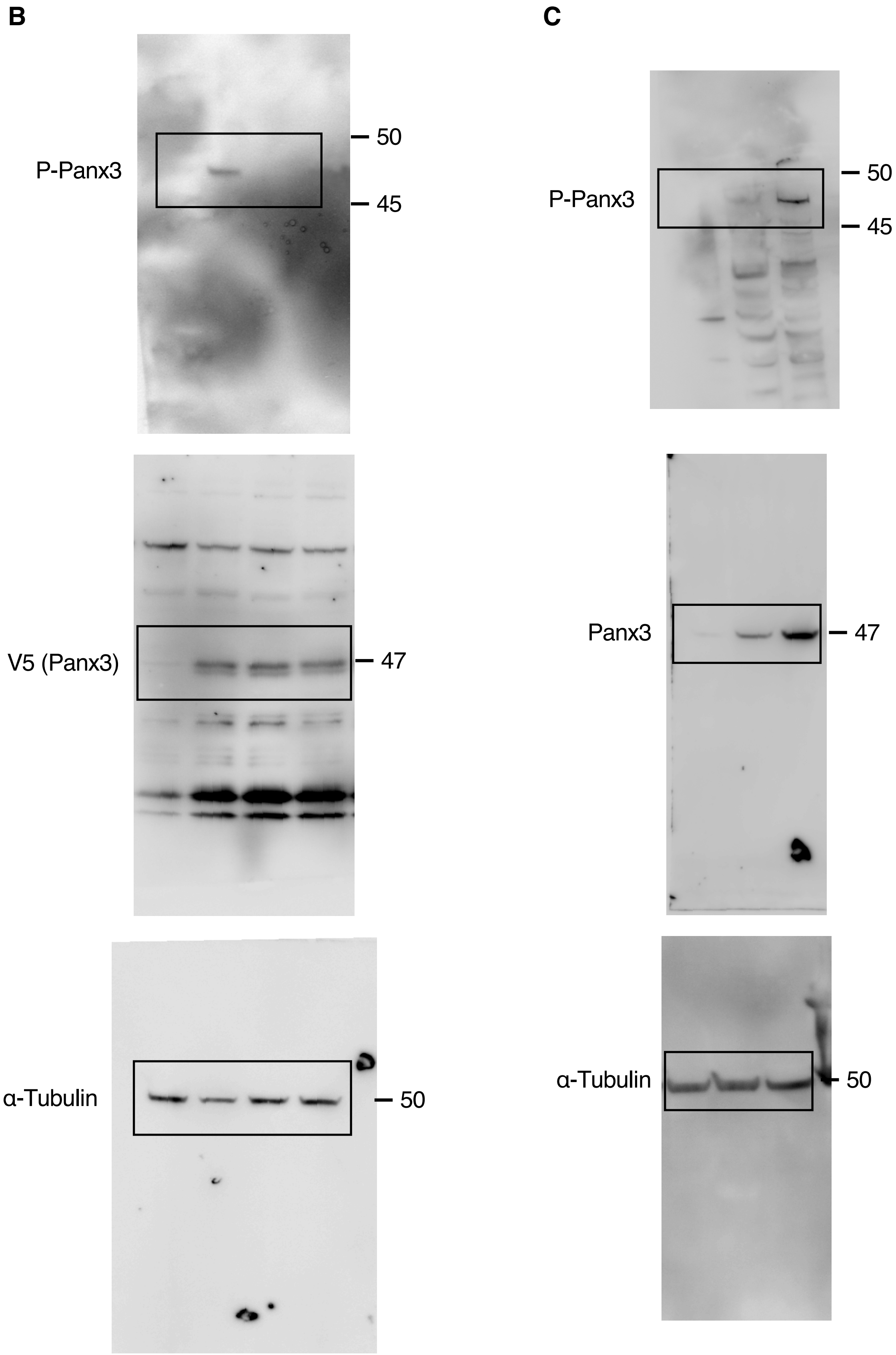

B
P-Panx3
50
45
V5 (Panx3)
47
α-Tubulin
50
C
P-Panx3
50
45
Panx3
47
α-Tubulin
50

IV

A

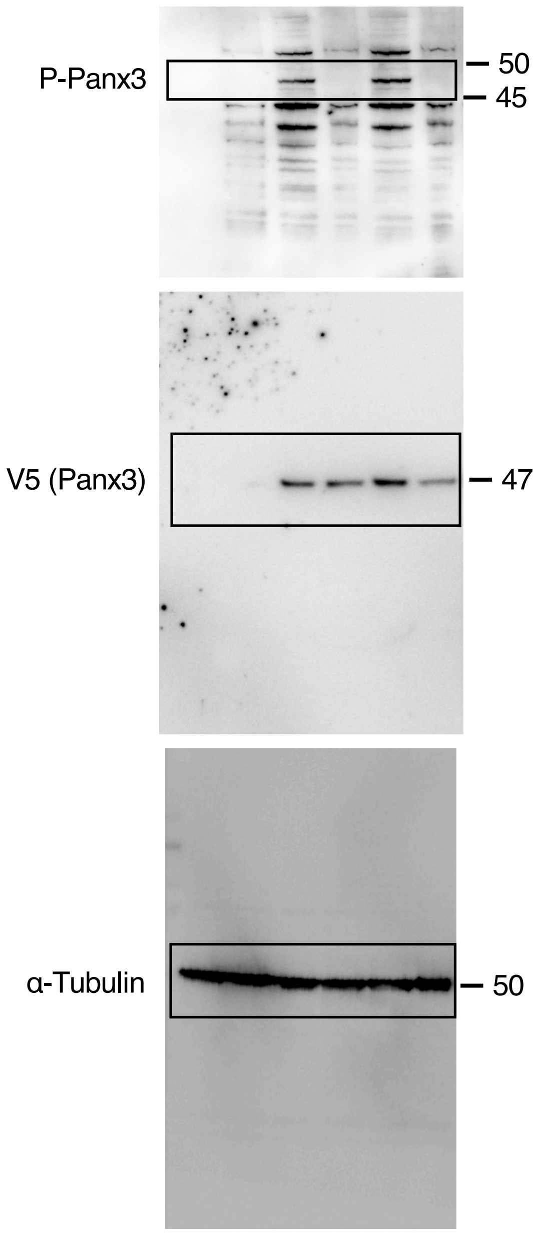

B

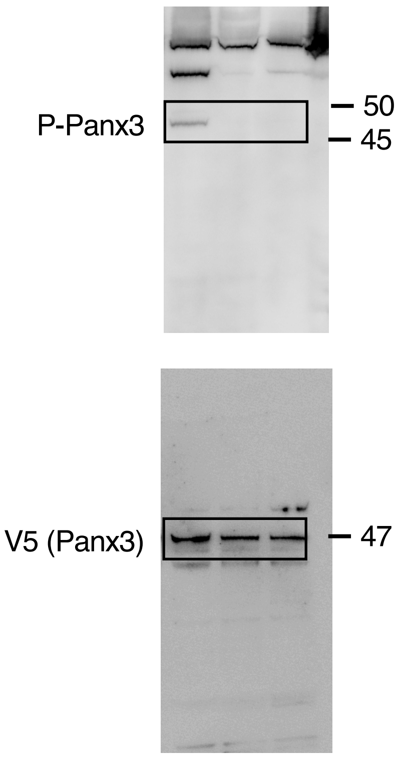

IV

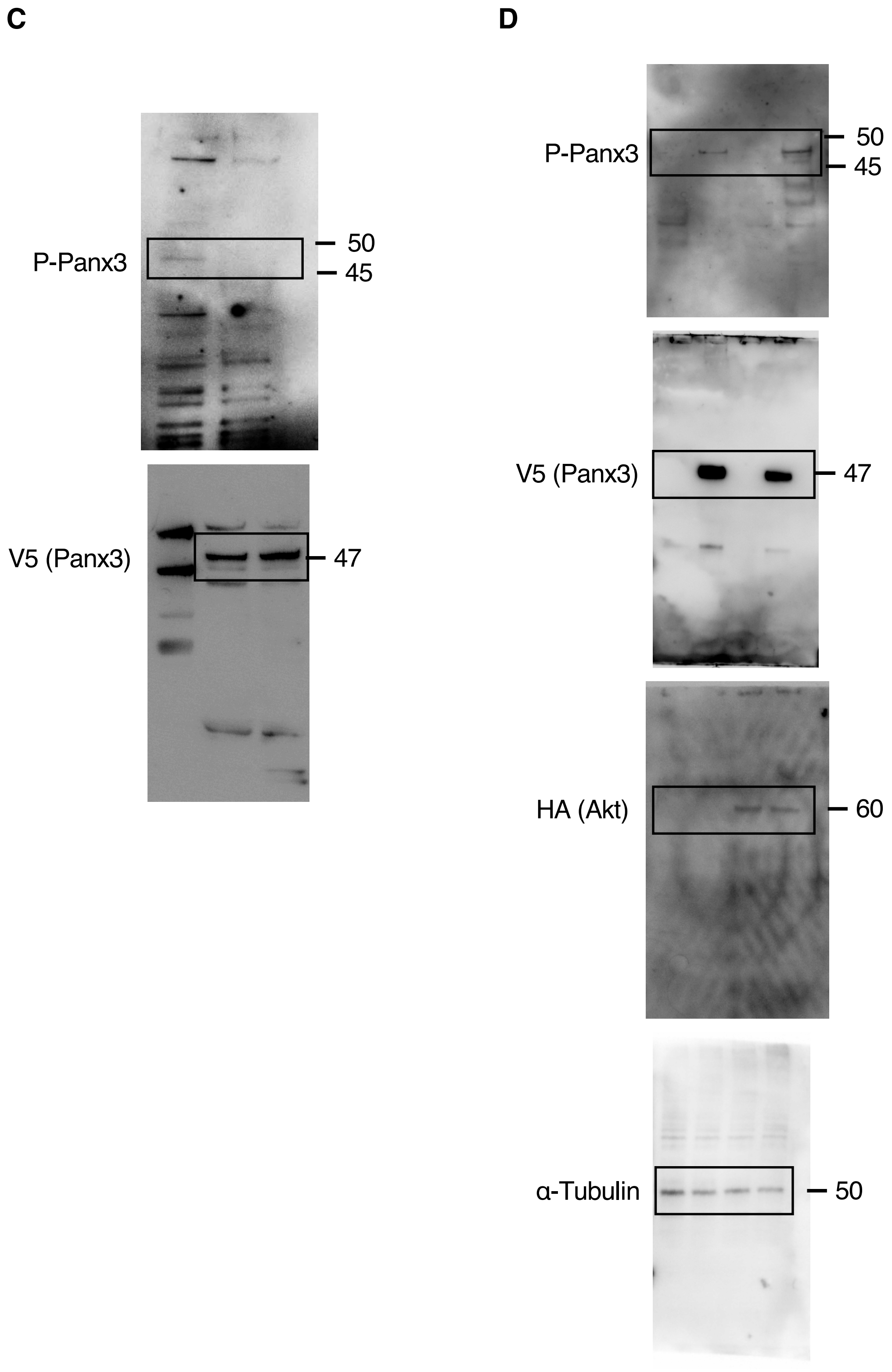
C
P-Panx3
50
45
V5 (Panx3)
47
D
P-Panx3
50
45
V5 (Panx3)
47
HA (Akt)
60
α-Tubulin
50

IV

E

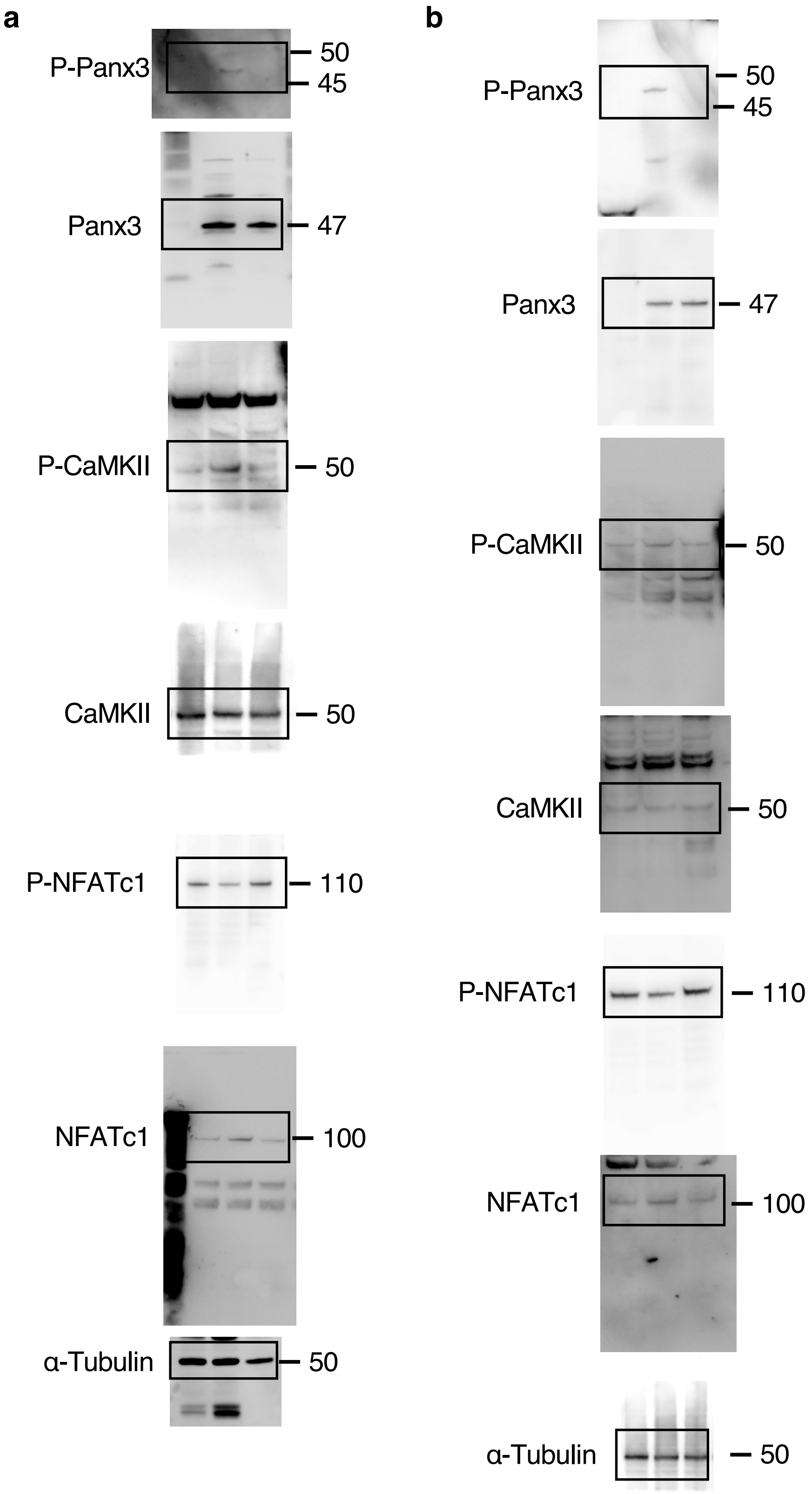
a
P-Panx3
50
45
Panx3
47
P-CaMKII
50
CaMKII
50
P-NFATc1
110
NFATc1
100
α-Tubulin
50
b
P-Panx3
50
45
Panx3
47
P-CaMKII
50
CaMKII
50
P-NFATc1
110
NFATc1
100
α-Tubulin
50
